# Association between maternal-child interaction, maternal depression and early child development: an observational sub-study in rural Zimbabwe

**DOI:** 10.1136/bmjpo-2025-003934

**Published:** 2026-05-17

**Authors:** Yujing Ooi, Jaya Chandna, Florence D Majo, Naume V Tavengwa, Batsirai Mutasa, Gwendoline Kandawasvika, Bernard Chasekwa, Jean H Humphrey, Robert Ntozini, Andrew J Prendergast, Melissa Gladstone

**Affiliations:** 1 Department of Women and Children’s Health, University of Liverpool Institute of Life Course and Medical Sciences, Liverpool, England, UK; 2 Zvitambo Institute for Maternal and Child Health Research, Harare, Zimbabwe; 3 Department of Paediatrics and Child Health, University of Zimbabwe, Harare, Zimbabwe, Zimbabwe; 4 Johns Hopkins Bloomberg School of Public Health, Baltimore, Maryland, USA; 5 Queen Mary University of London Blizard Institute, London, England, UK

**Keywords:** Infant, Low and Middle Income Countries, Mothers, Caregivers, Depression, Postpartum

## Abstract

**Introduction:**

Maternal responsiveness, defined as a mother’s ability to perceive her infant’s behaviour and respond appropriately, may be compromised with maternal depression and thereby impact early child development (ECD). These relationships have not been studied comprehensively in sub-Saharan Africa, where two-thirds of children are at risk of not reaching their developmental potential. This sub-study of the Sanitation Hygiene Infant Nutrition Efficacy (SHINE) trial in rural Zimbabwe evaluated the relationship between maternal-child interaction, maternal depressive symptoms and ECD using validated tools.

**Methods:**

The Edinburgh Postnatal Depression Scale (EPDS) and Observed Mother-Child Interaction (OMCI) tool were administered to mother-infant dyads in the cluster-randomised trial. ECD was assessed using the Malawi Developmental Assessment Tool (MDAT) and MacArthur-Bates Communicative Development Inventory (CDI). Generalised estimating equations were used to estimate effect sizes while accounting for within-cluster correlation and primary results were adjusted for confounding factors.

**Results:**

A total of 540 mother-infant dyads were included. 5.5% of mothers met the EPDS cut-off score for depressive symptoms. There was a positive association between OMCI score and MDAT motor, language and MacArthur-Bates CDI. For every 10 unit change in total OMCI score, there was a rise of 5.1 units (95% CI 3.6 to 6.6) in total MDAT score (out of 138 points), equivalent to a 0.54 SD increase. There was no statistically significant association between EPDS score and OMCI or ECD scores.

**Conclusion:**

Within a sample of rural Zimbabwean mother-infant dyads undergoing directly observed assessment, quality of maternal-child interaction was associated with ECD scores at 24 months of age.

**WHAT IS ALREADY KNOWN ON THIS TOPIC**

**WHAT THIS STUDY ADDS**

- In a sample of infants from a low-middle income country, quality of maternal-child interaction was associated with early child development scores at 24 months of age.

**HOW THIS STUDY MIGHT AFFECT RESEARCH, PRACTICE OR POLICY**

- Maternal-child interaction may represent a key area to target to improve early child development outcomes in low-middle income countries. Culturally validated tools, such as the Observation of Mother-Child Interaction (OMCI) tool, should be used more widely in low-middle income settings to provide an objective assessment of maternal-child interaction in future research.

## Introduction

 It is increasingly recognised that a nurturing environment with responsive caregiving alongside elements such as nutrition, sanitation and access to healthcare forms the foundation for development of the child. Maternal responsiveness, defined as the mother’s ability to perceive her child’s behaviour and respond promptly and appropriately, has been linked to improved social and emotional development, cognitive development and physical health.^1^ In line with this, the WHO, UNICEF and the World Bank have developed the Nurturing Care Framework, which calls for scale-up of ECD interventions, including those focusing on stimulation and responsive caregiving.^2^ It has been demonstrated that interventions enhancing maternal-child interaction may improve ECD outcomes in domains such as cognition, language, socioemotional and behaviour.^3^

Despite this knowledge, there is a paucity of data on maternal-child interaction, what influences better interactions and how it relates to child outcomes such as child development in low- and middle-income countries (LMICs), especially in sub-Saharan Africa.^4^ This gap is made more critical by the fact that infants in LMICs often fail to reach their full potential, and may therefore be more likely to benefit from such interventions due to reduced access to nurturing care.^5^ In several small interventional studies, there appears to be a correlation between interventions to improve maternal-child interaction and better early child development outcomes in sub-Saharan African populations, which emphasises the importance of understanding this relationship further and on a larger scale.^6^

A lack of validated culturally sensitive tools and methodology to measure maternal-child interaction in sub-Saharan Africa may contribute to this gap in the literature. Mothers in rural non-Western societies show differences in maternal responsiveness and caregiving behaviours in comparison to Western mothers, which calls for the development and use of validated culturally sensitive tools.^4^

Maternal depression may also be an important determinant in child development. Proposed mechanisms include antenatal biological effects such as fetal exposure to elevated maternal cortisol as well as postnatal influences including interparental conflict.^7^ However, the strongest body of evidence relates to disruptions in parenting behaviours. Meta-analytic findings demonstrate that maternal depression is associated with reduced maternal responsiveness, a key component of early caregiving environments.^8^ The burden of postnatal depressive symptoms is reported to be higher in mothers in LMICs in comparison to high-income countries, underscoring the importance of examining the role of maternal depression in ECD within the LMIC contexts.^7^ In a meta-analysis of perinatal mental health disorders in African mothers, the prevalence of depression was around 18%,^9^ in keeping with systematic reviews and meta-analyses of perinatal depression in LMICs, showing postpartum depression affected around 1 in 5 women.^10^ Despite this knowledge, studies indicating an association between maternal depression and ECD outcomes are again largely focused in high-income countries.^11 12^ Evidence from a limited number of studies conducted in LMICs suggests an association between maternal symptoms of depression and poorer child developmental outcomes; however, effect sizes are generally small and there is a paucity of data from sub-Saharan Africa.^11 13^ This includes a study in Ethiopia which reported an association between maternal mental health symptoms and child development scores at 2 to 24 months of age.^14^ Other studies from LMICs have demonstrated similar associations with overall early child development scores or with specific developmental domains including social and motor functioning.^15^ One study in India demonstrated a relationship between maternal depressive symptoms and mental development quotients at 6 to 12 months of age, an effect partially mediated by parental responsiveness.^16^ Some studies have examined associations with behavioural outcomes suggesting increased risks of childhood behavioural problems and aggression, including evidence from South Africa.^17 18^

Overall, there remains a lack of large studies investigating the relationship between maternal depression, maternal-child interaction and ECD in sub-Saharan Africa.^13^ The SHINE trial was a large 2×2 factorial cluster-randomised trial assessing the individual and combined effects of improved infant and young child feeding (IYCF) and improved water, sanitation and hygiene (WASH) on child stunting, anaemia and ECD.^19^ The current sub-study aimed to evaluate the association between maternal-child interaction, maternal depressive symptoms and ECD scores using the Observation of Mother-Child Interaction (OMCI) tool and the Malawi Developmental Assessment Tool (MDAT; assessing motor, cognitive, language and social development), both of which have been validated for LMIC settings.^20 21^ We hypothesised that higher OMCI scores would be associated with higher ECD scores, while higher maternal depressive symptoms would be associated with lower ECD scores.

## Methods

### Study population

The study area comprised two rural districts of central Zimbabwe divided into 212 clusters. Each cluster was defined as the catchment area of 1–4 village health workers (VHW) employed by the Zimbabwean Ministry of Health and Child Care. Between 22 November 2012 and 27 March 2015, VHWs prospectively identified pregnancies and referred women to SHINE research nurses who recruited pregnant women based on eligibility criteria. Women were eligible if they permanently resided in a study cluster and were confirmed pregnant. The sample size for the SHINE trial was calculated to detect effect of the trial interventions on ECD outcomes and is previously reported, the sub-study of the SHINE trial reported here is exploratory.^22^

### Data collection in SHINE trial

At baseline, information regarding maternal characteristics, maternal depressive symptoms and household characteristics was collected by research nurses during home visits. At baseline, women were also tested for HIV using a rapid test algorithm; any women living with HIV were urged to seek immediate antenatal care for PMTCT interventions. Infant birth date, weight, sex and delivery details were transcribed from health facility records. All participants were scheduled to receive 15 VHW visits between enrolment and 12 months postpartum, monthly visits between 13 and 17 months postpartum and a review at 18 months postpartum for intervention implementation. Research nurses separate to the VHWs made home visits at 32 gestational weeks and at 1, 3, 6, 12, 18 months after birth to repeat data collection. At the 32 gestational weeks visit, women who tested HIV-negative at baseline were retested. At the 18 month visit, women who were HIV-negative through pregnancy were retested.

### Data collection in maternal-child interaction sub-study

To be eligible for the maternal-child interaction sub-study, children had to be eligible for the ECD sub-study, which required children to complete the 18 month visit and turn 24 months old (allowable age window 102–112 weeks) between 1 March 2016 and 30 April 2017. Baseline characteristics between participants recruited for the ECD sub-study, participants who were not eligible for the ECD sub-study, and those who were eligible but not recruited into the ECD sub-study have been previously reported.^22^ For the final analysis, children scoring moderate-severe on the Washington score for disability or not completing full maternal-child interaction assessment with maternal and child components were not included. Baseline characteristics between participants who then went on to be included in this sub-study measuring maternal-child interaction were compared with those not included.

At 24 months, a home visit was conducted by a research nurse to collect data on maternal depressive symptoms score, maternal-child interaction scores, early child development scores, disability scores, family care indicator scores, mother and child behaviour scores and anthropometry.

### Measures and tools

#### Maternal depressive symptoms

Maternal depressive symptoms were measured using the Edinburgh Postnatal Depression Scale (EPDS) administered by research nurses at the baseline visit (during pregnancy) and at 24 months postpartum. The EPDS is a 10-question self-report measure designed to screen women for perinatal depression and is the most extensively validated tool to screen for this.^23 24^ While the scale was designed for the postnatal period, it has been shown in study to be useful for screening for depression both in adults and in women who are not in the perinatal period and have older children.^25 26^ A cut-off of ≥12 at either time point was used to indicate risk of depression, as this has been validated in Zimbabwe against DSM IV criteria for depression using the Shona version of EPDS.^27^

### Maternal-child interaction

Maternal-child interaction was measured by research nurses at a study visit at age 24 months using the Observed Mother-Child Interaction (OMCI) tool, which uses a 5-min live observed interaction where the mother is handed a picture book, asked to show their child the book and play as they would normally. A trained assessor uses this tool to score 19 behavioural items (12 mother, 6 child and 1 shared). The number of times each behaviour is observed is recorded and used to create a composite score that rates the responsiveness of the interaction. Maternal behaviours assessed include affect, touch, eye contact, verbal statements and language stimulation; child behaviours assessed include affect, eye contact, communication and attention. The tool was developed on a rural population in Pakistan and has been previously used in Rwanda and in other studies in Zimbabwe.^2128^,^30^ It shows good reliability, internal consistency and inter-rater reliability, and has been validated for use in LMIC settings.^21^

The child component of the score was introduced first, followed by the maternal component later in the study protocol. Analysis was only carried out on the sub-set of dyads that had both infant and maternal scores performed.

### Family care indicators

Stimulation in the home was measured by research nurses at the 24 month study visit. Derived from Home Observations for Measurement of the Environment, which is a validated instrument for measuring learning opportunities and stimulation available in the home environment, UNICEF created the Family Care Indicators (FCI) as a shorter tool that is easier to administer on a large scale across different cultural settings.^31 32^

#### Food insecurity

A version of the coping strategies index was used at the 24 month study visit as a proxy measure for food insecurity.^33^ The full index consists of questions around frequency and severity of coping strategies used by a household over the past 30 days when they lack sufficient food. The questions are selected and weighted for the cultural context to produce a numerical score, with higher scores indicative of higher food insecurity. In this study a ‘reduced’ version of the index was administered, consisting of five questions, which is shown to reflect food insecurity with similar performance to the full index within Sub-Saharan Africa.^34^

### Early child development

Assessment of ECD was carried out at the home visit using the following tools, which were translated, back translated and preliminarily adapted for use in Zimbabwe, as previously described.^22^

### Malawi developmental assessment tool (MDAT)

This tool directly assesses child development in four domains: gross motor coordination (36 items), fine motor coordination (36 items), language (36 items), social (30 items). Fine motor, language and social domains also measure components of cognitive development. The MDAT was developed on a rural population in Malawi and has been validated for use in sub-Saharan Africa.^20^

### MacArthur-Bates communicative development inventory (CDI)

This is a specific assessment of child language according to maternal report, which includes a vocabulary and grammar checklist.^35^ For this sub-study, this test was formally adapted for Shona speakers using a rigorous method to produce a detailed protocol approved by the CDI team.

### Validation

All research nurses underwent a 3 week training programme, which included practical assessments that were double-marked by the gold-standard trainer using non-SHINE children, with percentage agreement between scores set at >85% to pass the training. This was repeated at 6-monthly intervals throughout the data collection process. In addition, 5% of all assessments were video-recorded and double-marked by a psychologist with expertise in these assessment tools (JC) and a neurodevelopmental paediatrician (GK). Percentage agreement was 94% for MDAT and 87% for the OMCI scores.

### Statistical methods

Analysis was carried out in Stata (V. 14.1). Children scoring moderate-severe on the Washington score for disability or without full maternal-child interaction assessment were removed. Primary analysis looked at the association between OMCI and ECD scores. Maternal-child interaction scores have been considered as the total combined score, maternal score only and infant score only. This is to explore if there might be any difference in effect of maternal and child behaviours on ECD.

The outcomes were total MDAT score, MDAT gross motor score, MDAT fine motor score, MDAT language score, MDAT social score, MacArthur-Bates CDI vocabulary checklist. Secondary analyses explored whether there was any association between EPDS score and OMCI score, or between EPDS score and ECD outcomes.

A generalised estimating equations approach was used to account for within-cluster correlation to estimate effect sizes. Confounders were considered by undertaking adjusted analyses using selected covariates in a multivariable regression model. The covariates included were trial factors (trial arm, study nurse and calendar month); maternal factors (maternal age, maternal education and HIV status in pregnancy); infant factors (child sex, birth weight and child age) and environmental factors (family care indicator score and wealth quintile). The confounders were identified from Direct Acyclic Graphs^36^ (see online supplemental figures 1–3). These factors were assessed first in bivariate analyses as possible confounding factors (using p<0.2 for continuous outcomes or difference >0.25 SD) using multinomial and ordinal regression models with robust variance estimation and Somers’ D for medians. Potential confounders assessed but not included in the final model included parity, disability and food insecurity (Coping Strategies Index).

Interaction testing was undertaken using a generalised estimating equations approach to determine if there was any interaction effect of HIV status in pregnancy and randomised trial arm (using p<0.05, Wald test). If interaction was present, stratified analyses were performed.

### Patient involvement

This trial was originally carried out from 2012 to 2015, and this sub-study is secondary data analysis using available data. The Shurugwi Community Advisory Board had input during design and implementation of the SHINE trial as well as in dissemination of trial results. Videos were created and shared with village health workers to aid in dissemination.

### Trial oversight

The study protocol, with the sub-study protocol included as an amendment, was approved by the Medical Research Council of Zimbabwe and the Institutional Review Board of the Johns Hopkins Bloomberg School of Public Health (Zimbabwe: MRCZ/A/1675; Johns Hopkins University: IRB#4205). The design and methods have been reported previously and the study protocol and prespecified statistical analysis plan can be found at https://osf.io/w93hy.^37^ Mothers provided written informed consent in local languages. The trial is registered at ClinicalTrials.gov NCT01824940.

## Results

**Figure 1:**
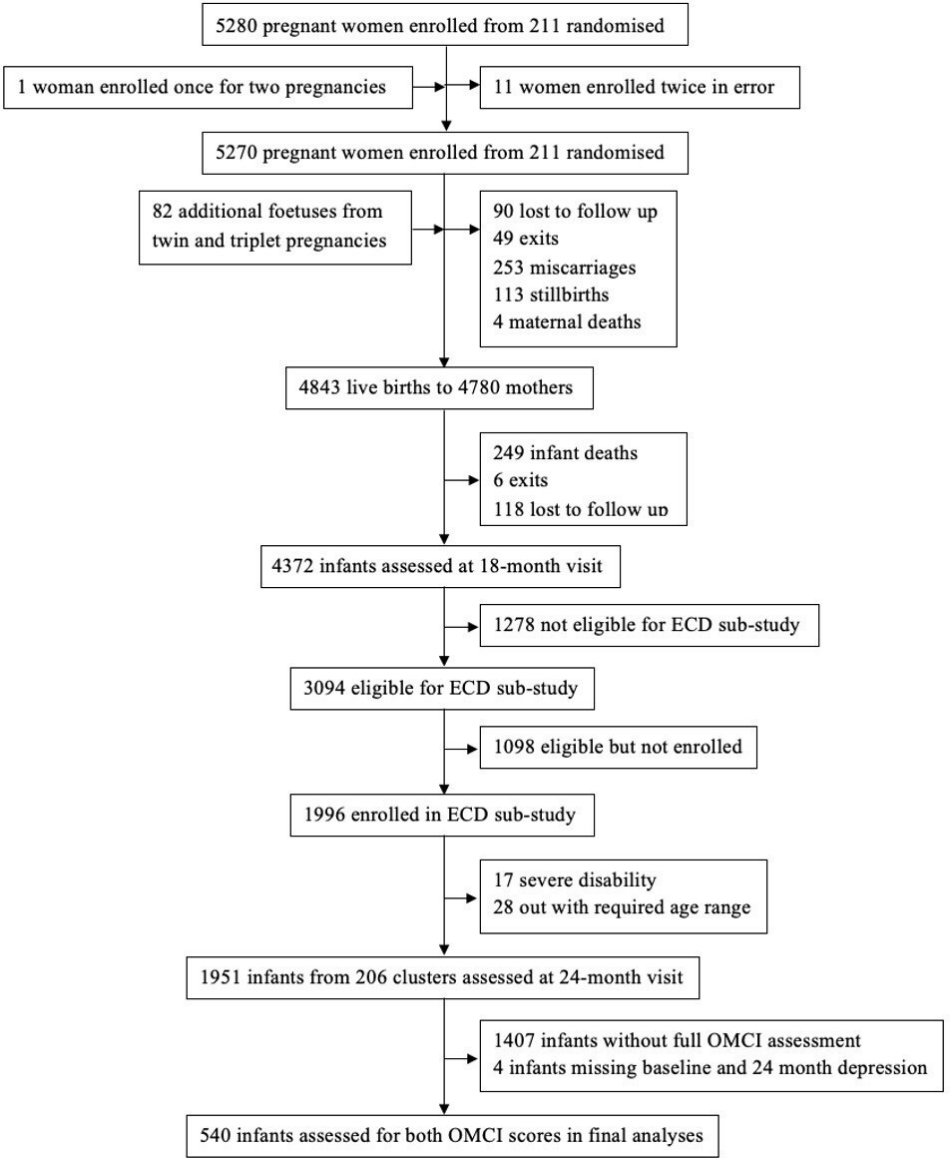
Flowchart of participants in the SHINE early child development sub-study (separate file). ECD, early child development; OMCI, observed mother-child interaction; SHINE, sanitation hygiene infant nutrition efficacy

A total of 5270 pregnant women were recruited from 211 clusters at a median gestational age of 12 weeks (IQR 16–19 weeks). During the study, there were 252 miscarriages, 4 maternal deaths, 113 stillbirths, 249 child deaths, 208 who were lost to follow-up or moved away from Zimbabwe and 55 who voluntarily left the study. Therefore, 4372 children were assessed at the 18 month visit. 3094 children became 102 to 112 weeks of age during the recruitment period, making them eligible for the ECD sub-study at 24 months. Of these, 1098 could not participate due to moving away from their study home, being unreachable by phone or home visit, or being unable to participate during the required age window. Therefore, a total of 1996 children were recruited into the ECD sub-study from 206 clusters and assessed for ECD scores at 24 months of age; 17 children were subsequently excluded because they had moderate to severe disability on screening using the Washington tool, and 28 children were found to be outside the required age window and so were removed from analysis. Of the 1951 children assessed, 540 completed the full OMCI assessment with maternal and infant score assessment and were therefore included in the analysis (figure 1).

Mothers included in the analysis were mostly educated and married, with only a few being employed (table 1). 47.6% of children included in the analysis were female, with an average birth weight of 3.1 kg. Infants were mostly born by vaginal delivery in institutions. 15.8% of mothers were living with HIV during pregnancy; 87.1% of these mothers were confirmed to be taking antiretroviral therapy. The baseline characteristics of mothers and infants eligible for the ECD sub-study but not enrolled were similar, and reported elsewhere.^35^ The baseline characteristics between mothers and infants included in the current analysis were similar compared with those eligible but not included (online supplemental table 1).

**Table 1:** Characteristics of participants in the sub-study at baseline during pregnancy

Baseline characteristic	
Women assessed, n	537
Children assessed, n	540
*Household characteristics*	
Size, median (IQR)(n)	4 (3, 6)(533)
Wealth quintile, percent(n)	
1 Lowest	16.0(80)
2 Lower middle	22.4(112)
3 Middle	21.8(109)
4 Upper middle	21.0(105)
5 Highest	18.6(93)
Electricity	
Power grid, percent(n)	3.2(16)
Other power, percent(n)	
Generator	2.6(13)
Solar	72.0(360)
No electricity	25.4(127)
*Diet quality and food security*	
Household meets minimum dietary diversity score, percent(n)	42.3(189)
Coping Strategies Index, median (IQR)(n)	0 (0, 5)(490)
*Maternal characteristics*	
Age (y), mean (SD)(n)	26.8 (6.8)(499)
Height (cm), mean (SD)(n)	159.5 (10.6)(522)
Completed schooling (y), mean (SD)(n)	9.6 (1.8)(529)
Parity, mean (SD)(n)	1.8 (1.5)(420)
Married, percent(n)	93.9(494)
Employed, percent(n)	8.6(41)
Study arm, percent(n)	
SOC	25.2(135)
IYCF	25.7(138)
WASH	24.1(130)
WASH+IYCF	25.0(134)
Depression at baseline, percent(n)	4.4(22)
Depression at 24 months, percent(n)	1.3(7)
Depression at baseline or 24 months, percent(n)	5.5(27)
Living with HIV during pregnancy, percent(n)	15.8(85)
Antiretroviral therapy	
Taking antiretroviral therapy, percent(n)	87.1(74)
Not taking antiretroviral therapy, percent(n)	0(0)
Missing antiretroviral therapy status, percent(n)	13.0(11)
*Child characteristics*	
Female, percent(n)	47.6(257)
Birth weight (kg), mean (SD)(n)	3.1 (0.4)(523)
Birth weight<2500 g, percent(n)	9.8(51)
Institutional delivery, percent(n)	89.8(466)
Vaginal delivery, percent(n)	94.1(496)
Study arm, percent(n)	
SOC	25.2(136)
IYCF	25.7(139)
WASH	24.1(130)
WASH+IYCF	25.0(135)

IQR, interquartile range; IYCF, Infant and Young Child Feeding; MUAC, Mid-upper arm circumference; SD, standard deviation; SOC, Standard of Care; WASH, Water and Sanitation/Hygiene.

The results of the multivariable analysis of OMCI score and ECD outcomes are presented in table 2. There was a positive association between OMCI score and ECD score in MDAT total, MDAT fine motor, MDAT gross motor, MDAT language and MacArthur-Bates CDI domains. There was the greatest evidence for a relationship with the MDAT language score, where for every 10-unit change in total OMCI score, there was a rise of 2.4 units (95% CI 1.7 to 3.1) in MDAT language score (out of 36 points), equivalent to a 0.54 SD increase. In the MacArthur-Bates CDI score, for every 10-unit change in total OMCI score, there was an increase of 9 words (95% CI 6.04 to 11.91) in MacArthur-Bates CDI score (out of 99 potential words), equivalent to a 0.45 SD increase. The point estimates were larger when using the infant component of the OMCI score, in comparison to the maternal or total OMCI score. The only domain that did not show this positive association was the MDAT social domain.

**Table 2:** OMCI scores (independent) and ECD scores (dependent) at 24 months of age

ECD score	OMCI score type	Coefficient (95% CI)	p-value
Malawi Developmental Assessment Tool (MDAT) (n=511)			
Total score	Maternal	0.61 (0.38, 0.83)	<0.001
	Infant	0.77 (0.52, 1.03)	<0.001
	Total	0.51 (0.36, 0.66)	<0.001
Fine motor	Maternal	0.14 (0.07, 0.21)	<0.001
	Infant	0.19 (0.12, 0.27)	<0.001
	Total	0.12 (0.07, 0.17)	<0.001
Gross motor	Maternal	0.17 (0.08, 0.25)	<0.001
	Infant	0.18 (0.09, 0.26)	<0.001
	Total	0.13 (0.08, 0.18)	<0.001
Language	Maternal	0.26 (0.16, 0.36)	<0.001
	Infant	0.39 (0.27, 0.51)	<0.001
	Total	0.24 (0.17, 0.31)	<0.001
Social	Maternal	0.03 (-0.03, 0.07)	0.377
	Infant	0.02 (-0.05, 0.09)	0.582
	Total	0.02 (-0.02, 0.05)	0.391
MacArthur-Bates CDI (n=505)			
	Maternal	1.00 (0.52, 1.48)	<0.001
	Infant	1.45 (0.89, 2.00)	<0.001
	Total	0.90 (0.60, 1.19)	<0.001

Adjusted for trial arm, study nurse, calendar month of birth, maternal age, maternal education, HIV status in pregnancy, wealth quintile, child sex, birth weight, family care indicator score and child age.

CDI, Communicative Development Inventory.ECD, early child development; OMCI, Observation of Mother-Child Interaction.

Adjusted multivariable analysis of OMCI score in quartiles and ECD scores was carried out, which demonstrates the association between higher total and individual domain MDAT score with higher total OMCI score quartile, except for in the social domain (online supplemental tables 2,3). Interaction analysis was carried out demonstrating no interaction by trial arm and HIV status in pregnancy. ICYF arm and HIV positive status (p=0.85), ICYF arm and HIV negative status (p=0.80), WASH arm and HIV positive status (p=0.93), WASH arm and HIV negative status (p=0.64). As there was no interaction, stratified analyses were not carried out.

There was no clinically significant association found between EPDS score and OMCI or ECD scores at 24 months of age (table 3).

**Table 3:** Maternal depression (independent) and OMCI score or ECD score (dependent) at 24 months of age

OMCI score type (dependent)	Coefficient	95% CI		P-value
Maternal	−0.237	−1.115	0.641	0.596
Infant	−0.547	−1.362	0.267	0.187
Total	−0.785	−2.250	0.680	0.293
**ECD outcome (dependent**)	**Coefficient**	**95% CI**		**P-value**
MDAT total	−1.796	−4.075	0.481	0.122
Fine motor	−0.527	−1.087	0.033	0.065
Gross motor	−0.379	−1.151	0.392	0.335
Language	−0.730	−1.792	0.332	0.177
Social	−0.160	−0.739	0.419	0.588
MacArthur-Bates CDI	−4.013	−8.828	0.801	0.102

Exposure variable was Edinburgh Postnatal Depression Score (EPDS)>=12 at 12 or 24 month visit.

Adjusted for trial arm, study nurse, calendar month of birth, maternal age, maternal education, HIV status in pregnancy, wealth quintile, child sex, birth weight, Family Care Indicator score and child age.

CDI, Communicative Development Inventory.ECD, early child development; MDAT, Malawi Developmental Assessment Tool; OMCI, Observation of Mother-Child Interaction.

## Discussion

An estimated 250 million children (43%) under 5 years of age in LMIC settings are at risk of not meeting their development potential.^5^ Our study evaluated the association between quality of maternal-child interaction and ECD, demonstrating an association between higher quality of interaction and better early child development outcomes at 24 months of age after adjustment for confounding factors in a large cohort of children in rural Zimbabwe. This association was evident across the domains of development, except the social domain. The lack of association between maternal-child interaction and MDAT social domain may be due to this domain’s lack of sensitivity to change at the age of 24 months as well as its reliance on maternal report rather than observation. Many organisations already encourage maternal-child interaction through promotion of responsive caregiving practices (e.g. WHO/UNICEF Care for Child Development modules); however, few studies have demonstrated a measurable correlation between maternal-child interaction and early child development in LMIC settings.^4^ Conducting these studies in LMIC settings, where children may be at higher risk of reduced responsive caregiving but have different cultural caregiving practices, is of paramount importance.

Interestingly, this cohort did not demonstrate any association between maternal depressive symptoms and OMCI or early child development scores. While there is some previous literature demonstrating an association between maternal depression and reduced quality of maternal responsiveness and poorer early child development, the evidence is still unclear especially in LMIC settings due to small sample sizes and heterogenous studies.^13 38^ In our study, 5% of women screened positive for maternal depressive symptoms by meeting the cut-off criteria for EPDS score. It should be noted that this proportion was lower than in the overall SHINE cohort (online supplemental table 1). The lower proportion may be due to a lack of symptom endorsement in our setting or simply a low prevalence of maternal depressive symptoms in our sample. It should be noted that EPDS is not a diagnostic test for depression but a screening tool, and this may have influenced our findings.

### Strengths and limitations

The strengths of our study include the utilisation of a large cohort of children within the SHINE trial in rural Zimbabwe. Our study is one of few that has used simple tools to measure maternal-child interaction directly at the time of assessment in an African setting. This live observation may actually provide a more objective assessment than tools which use maternal report or videotaped interaction, as it has been shown in other settings that live observation results in a higher number of observed behaviours, possibly due to a better vantage point of the observer and less observer fatigue.^39^ There is also some evidence that use of cameras to videotape parent-infant interactions influences both infants and parent behaviours.^40^ Furthermore, we utilised a team of trained Zimbabwean assessors (rather than Western psychologists as is often the case) to make contemporaneous assessments on maternal-child interaction, which makes the tool more culturally relevant and applicable. We hope that our work will lead to future use of the OMCI on a wider scale in low-middle income settings, particularly in Africa. In addition, we used a culturally sensitive child development tool (MDAT), and we formally adapted the MacArthur Bates CDI in the local language, thereby improving the local relevance of our measures. In our data collection process, we ensured standardisation and regular quality checks. Finally, by adjusting for a number of prespecified covariates, we have increased the precision of our effect estimates.

There are a number of limitations in this study. We were only able to obtain OMCI data for 27% of the SHINE cohort, leading to the sample of 540 children in this sub-study. However, on comparing baseline characteristics to those in the trial without full OMCI scores, there do not appear to be any substantial differences, so we do not believe this is a major source of bias. Only a small proportion of women met depressive symptom criteria despite using a tool with a validated cut-off score for Zimbabwe. The proportion was smaller than expected looking at the literature in sub-Saharan Africa, and limitations of this include that the subgroup studied was not fully representative of the wider Zimbabwean population of women with young children and there may be under-powering in this study to detect any associations.^9^ Assessors of OMCI and MDAT/CDI were blinded to trial arm allocation but not to maternal EPDS score, as these were collected by the research nurse at the same study visit. We do not believe this has introduced substantial bias to our results as we had 87% (OMCI) and 94% (MDAT) percentage agreement in a proportion of assessments that were video recorded and double-marked by experts in use of these tools. Furthermore, in this study, we present outcomes at age 24 months, whereas testing of early child development at older ages may be more sensitive to differences in cognitive function which will persist into adulthood. Although we found a statistically significant relationship between maternal-child interaction and early child development at 2 years of age, the data remain observational and therefore it is not possible to comment on causation.

In conclusion, higher maternal-child interaction as measured by the OMCI was associated with better ECD scores at 2 years of age as measured by the MDAT, and better language scores as measured by the MacArthur-Bates CDI. It is possible that maternal responsiveness may represent a key area for intervention in LMIC settings to improve ECD outcomes. While interventions are available to improve maternal-child interaction, direct observation of interaction is often not measured on a large scale. We recommend future use of the OMCI tool in low middle income settings, particularly in Africa, to provide an objective measure of interaction. Further research is needed to advance understanding of the effect that maternal-child interaction has on ECD and longer term outcomes within similar cohorts.

## Supplementary material

10.1136/bmjpo-2025-003934 online supplemental file 1

## Data Availability

Data are available in a public, open access repository.
