# Supplementary material for: Association between maternal-child interaction, maternal depression and early child development: an observational sub-study in rural Zimbabwe
Source: BMJ Paediatr Open. 2026 May 17;10(1):e003934. doi: 10.1136/bmjpo-2025-003934 (PMC13182492; doi:10.1136/bmjpo-2025-003934)
Supplement: online supplemental file 1 [file bmjpo-10-1-s001.pdf]

Online supplementary materials

Supplementary Figure 1

Direct acyclic graph illustrating confounding factors– maternal child interaction and early child development

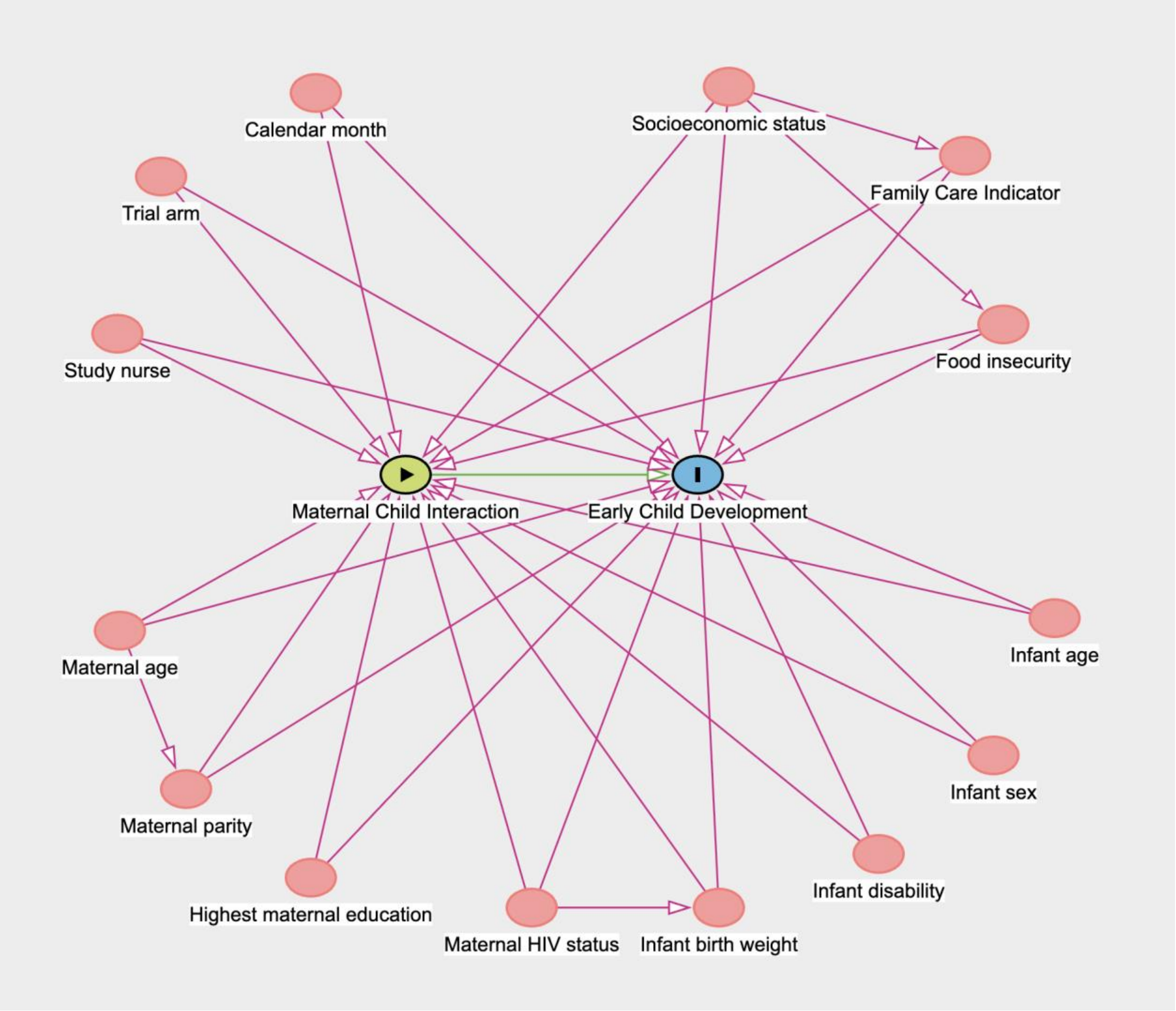

Supplementary Figure 2

Direct acyclic graph illustrating confounding factors – maternal depressive symptoms and early child development

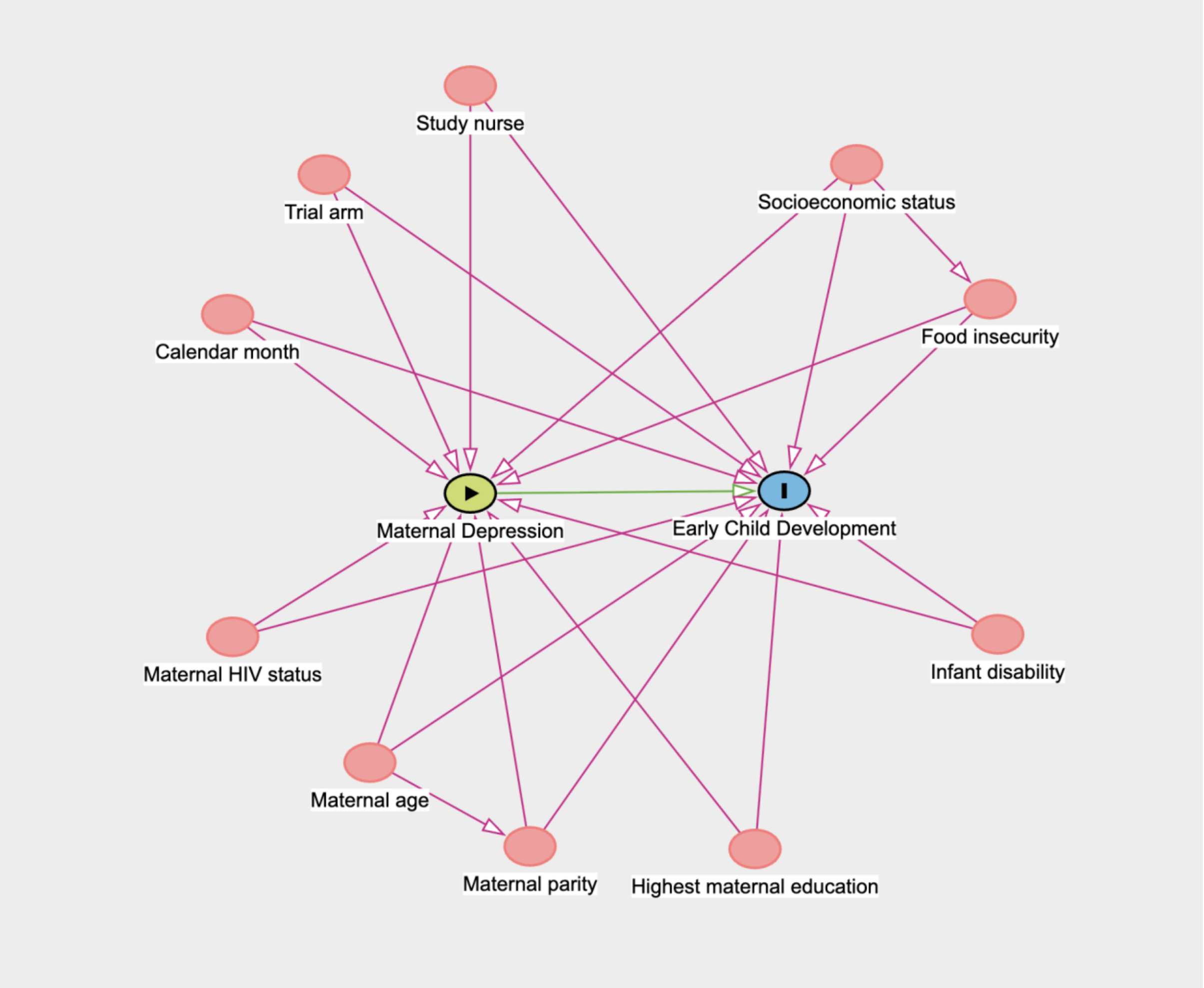

Supplementary Figure 3

Direct acyclic graph illustrating confounding factors – maternal depressive symptoms and maternal-child interaction

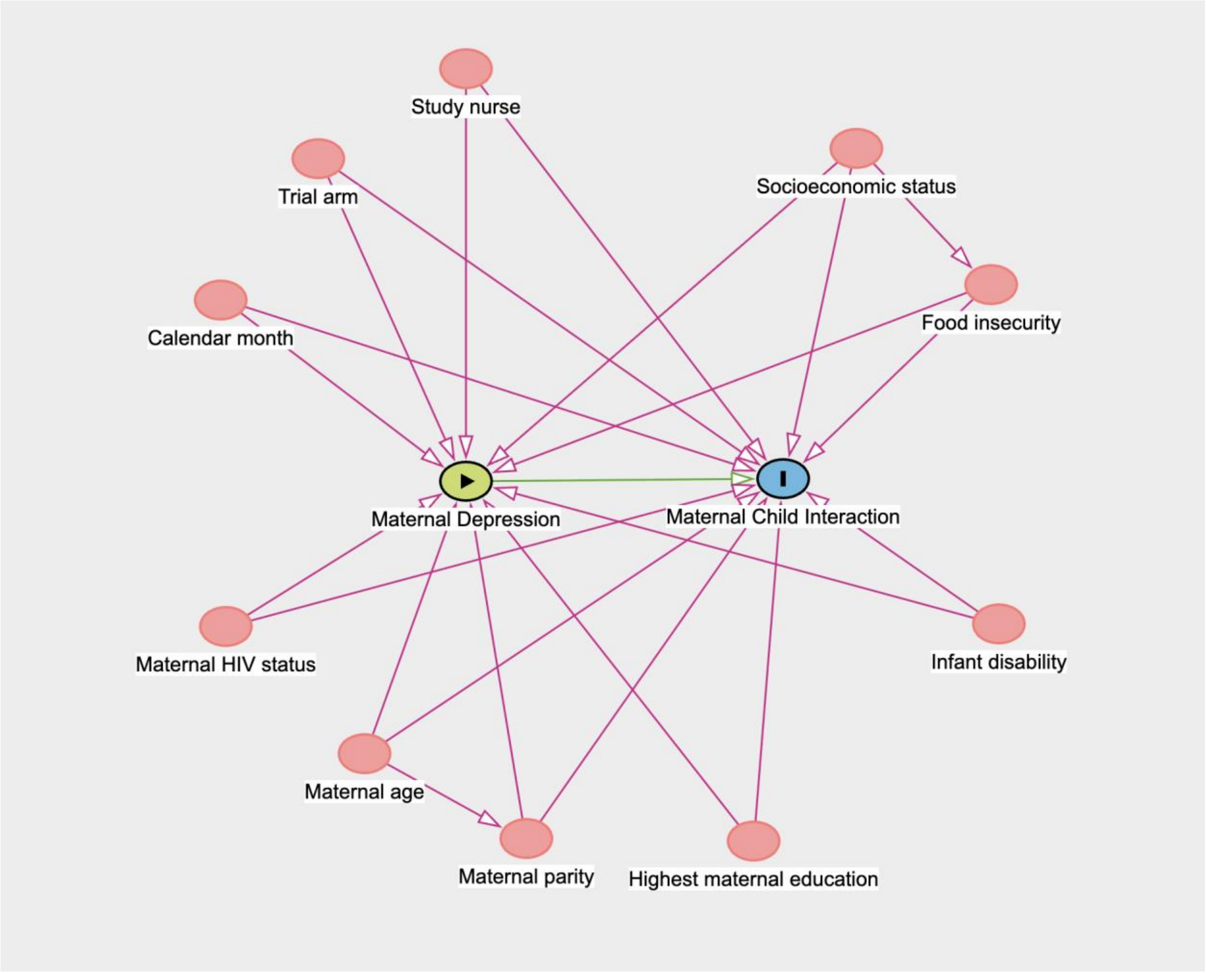

Supplementary Table 1

Characteristics of mothers and infants included and not included in the maternal-child interaction sub-study, at baseline visit during pregnancy

**Baseline characteristic**	**Eligible for sub-study with both OMCI scores**	**Eligible for sub-study without both OMCI scores**	**p-value**
Women assessed, N	537	1438	
Children assessed, N	540	1456	
Household characteristics			
Size, median (IQR) [n]	4 (3, 6) [533]	5 (3, 6) [1338]	0.46
Wealth quintile, percent [n]			0.27
1 Lowest	16.0 [80]	19.3 [262]	
2 Lower middle	22.4 [112]	19.0 [259]	
3 Middle	21.8 [109[	20.5 [278]	
4 Upper middle	21.0 [105]	20.8 [282]	
5 Highest	18.6 [93]	20.5 [278]	
Electricity			
Power grid, percent [n]	3.2 [16]	3.0 [40]	0.08
Other power, percent [n]			0.15
Generator	2.6 [13]	3.5 [48]	
Solar	72.0 [360]	67.5 [915]	
No electricity	25.4 [127]	29.0 [393]	
Diet quality and food security			

Household meets minimum dietary diversity score, percent [n]	42.3 [189]	40.7 [477]	0.55
Coping Strategies Index, median (IQR) [n]	0 (0, 5) [490]	2 (0, 9) [1324]	<0.001
Maternal characteristics			
Age (y), mean (SD) [n]	26.8 (6.8) [499]	27.3 (6.7) [1259]	0.16
Height (cm), mean (SD) [n]	159.5 (10.6) [522]	159.9 (8.8) [1407]	0.44
Completed schooling (y), mean (SD) [n]	9.6 (1.8) [529]	9.5 (1.8) [1329]	0.73
Parity, mean (SD) [n]	1.8 (1.5) [420]	2.1 (1.4) [1037]	<0.001
Married, percent [n]	93.9 [494]	96.3 [1272]	0.04
Employed, percent [n]	8.6 [43]	9.5 [128]	0.59
Study arm, percent [n]			0.23
SOC	25.1 [135]	22.4 [322]	
IYCF	25.7 [138]	23.2 [333]	
WASH	24.2 [130]	25.7 [369]	
WASH+IYCF	25.0 [134]	28.8 [414]	
Depression at baseline, percent [n]	4.4 [22]	4.8 [61]	0.89
Depression at 24 months, percent [n]	1.3 [7]	2.0 [28]	0.33
Depression at baseline or 24 months, percent [n]	6.2 [81]	5.5 [27]	0.60
Living with HIV in pregnancy, percent [n]	15.8 [85]	16.2 [233]	0.84

Anti-retroviral therapy			<0.001
Taking anti-retroviral therapy	87.1 [74]	85.4 [199]	
Not taking anti-retroviral therapy, percent [n]	0 [0]	5.6 [13]	
Missing anti-retroviral therapy status, percent [n]	13.0 [11]	9.0 [21]	
Child characteristics			
Female, percent [n]	47.6 [257]	51.1 [744]	0.14
Birth weight (kg), mean (SD) [n]	3.1 (0.4) [523]	3.1 (0.5) [1378]	0.47
Birth weight < 2,500g, percent [n]	9.8 [51]	8.5 [117]	0.37
Institutional delivery, percent [n]	89.8 [466]	88.8 [1215]	0.56
Vaginal delivery, percent [n]	94.1 [496]	92.2 [1297]	0.18
Study arm, percent [n]			0.18
SOC	25.2 [136]	22.3 [325]	
IYCF	25.7 [139]	22.9 [334]	
WASH	24.1 [130]	25.9 [377]	
WASH+IYCF	25.0 [135]	28.8 [420]	

IQR, interquartile range; SD, standard deviation; SOC, Standard of Care; MUAC, Mid-upper arm circumference; IYCF, Infant and Young Child Feeding; WASH, Water and Sanitation/Hygiene.

Supplementary Table 2

OMCI total score (maximum score 57) in quartiles for the 540 participants included in the ECD sub-study.

OMCI quartile	N	Mean	SD	p25	p50	p75
**1**	139	23.0	2.6	22	23	25
**2**	147	28.5	1.1	28	28	29
**3**	124	32.3	1.1	31	32	33
**4**	130	38.6	3.2	36	38	40
**Total**	540	30.4	6.1	26	30	34

Supplementary Table 3

Early child development outcomes (dependent) and OMCI score in quartiles (independent) at 24 months of age.

ECD outcome	OMCI quartile	Coefficient	95% confidence interval		P>|z|
Malawi Developmental Assessment Tool (n=511)					
Total score	Q2	3.400	1.480	5.320	0.001
	Q3	4.598	2.321	6.875	<0.001
	Q4	7.528	5.146	9.910	<0.001
Fine motor	Q2	0.637	0.039	1.236	0.037
	Q3	0.958	0.274	1.642	0.006
	Q4	1.500	0.808	2.192	<0.001
Gross motor	Q2	0.935	0.288	1.581	0.005
	Q3	1.482	0.752	2.211	<0.001
	Q4	1.967	1.189	2.745	<0.001
Language	Q2	1.238	0.309	2.167	0.009
	Q3	1.742	0.668	2.815	0.001
	Q4	3.554	2.428	4.680	<0.001
Social	Q2	0.530	-0.121	1.181	0.111
	Q3	0.333	-0.264	0.931	0.274
	Q4	0.351	-0.252	0.954	0.254
MacArthur-Bates CDI (n=505)					
	Q2	3.774	-0.552	8.101	0.087

	Q3	7.260	2.320	12.201	0.004
	Q4	12.130	7.560	16.700	<0.001

Adjusted for trial arm, study nurse, calendar month of birth, maternal age, maternal education, HIV status in pregnancy, wealth quintile, maternal depression, child sex, birth weight, Family Care Indicator score and child age
