# Supplementary material for: Association between maternal-child interaction, maternal depression and early child development: an observational sub-study in rural Zimbabwe
Source: BMJ Paediatr Open. 2026 May 17;10(1):e003934. doi: 10.1136/bmjpo-2025-003934 (PMC13182492; doi:10.1136/bmjpo-2025-003934)
Supplement: Supplementary file 2 [file bmjpo-2025-003934.reviewer_comments.pdf]

PEER REVIEW HISTORY

BMJ Paediatrics Open publishes all reviews undertaken for accepted manuscripts. Reviewers are asked to complete a checklist review form and are provided with free text boxes to elaborate on their assessment. These free text comments are reproduced below.

ARTICLE DETAILS

**TITLE (PROVISIONAL)**	The association between maternal-child interaction, maternal depression and early child development: an observational substudy in rural Zimbabwe
**AUTHORS**	Ooi, Yujing<br>Chandna, Jaya<br>Majo, Florence D.<br>Tavengwa, Naume V.<br>Mutasa, Batsirai<br>Kandawasvika, Gwendoline<br>Chasekwa, Bernard<br>Humphrey, Jean H<br>Ntozini, Robert<br>Prendergast, Andrew J.<br>Gladstone, Melissa

VERSION 1 – REVIEW

**REVIEWER NAME**	Maruyama, Jessica Mayumi
**REVIEWER AFFILIATION**	Mackenzie Presbyterian University
**REVIEWER CONFLICT OF INTEREST**	No Conflict of Interest
**AI DISCLOSURE**	I used ChatGPT (OpenAI, GPT-5) only to enhance the clarity and flow of my review text. The critical evaluation and all scientific judgments are entirely my own.
**DATE REVIEW RETURNED**	06-Oct-2025

**GENERAL COMMENTS**	Dear Editors and Authors,<br>Thank you for the opportunity to review this interesting and well-written manuscript. The study makes a valuable contribution to the literature on early child development and maternal mental health in low- and middle-income settings, using appropriate methodology and culturally adapted tools. My comments below are intended to further strengthen the clarity and interpretability of the paper.<br>This manuscript presents a well-designed and clearly written secondary analysis from the SHINE trial, investigating the associations among maternal-child interaction, maternal depressive symptoms, and early child development (ECD) in rural Zimbabwe. The study addresses a relevant gap in the literature, since evidence linking these domains in sub-Saharan Africa remains limited. The use of culturally adapted, validated instruments (OMCI, MDAT, and CDI) and the careful adjustment for confounders are important strengths. However, some sections would benefit from deeper interpretation, particularly regarding the lack of association with maternal depressive symptoms, and clarifications in the Methods and Results.<br>Major comments:<br>1. The Methods section mentions that a conceptual framework

	guided the identification of potential confounders (Supplementary Figure 1) and that variables were tested using bivariate models before inclusion in multivariable analyses. However, it remains unclear which covariates were actually retained in the final adjusted models shown in Tables 2 and 3. Although these variables are listed in the table footnotes, it would be preferable to describe them explicitly in the Methods section, along with a brief rationale for their inclusion. In addition, while the framework presented is conceptually sound, it appears to represent a conceptual rather than a formal causal model. The authors might consider discussing how a Directed Acyclic Graph (DAG) could be used to clarify hypothesised causal relationships between maternal depression, mother–child interaction, and ECD outcomes. This would help to distinguish true confounders from mediators or colliders and enhance the causal interpretability of the adjusted analyses.<br>2. The Coping Strategies Index is presented in Table 1 but not discussed elsewhere in the text. It would be useful to provide a brief description of how this index was constructed and scored, including the range of possible values, what higher scores indicate, and whether it reflects short-term or chronic food insecurity. Additionally, please explain whether this variable was considered as a potential confounder (e.g., proxy for household stress or food insecurity) in the adjusted analyses. Clarifying its role would improve readers' understanding of the socioeconomic context of the sample.<br>3. The paper focuses on EPDS measured at 24 months, yet depressive symptoms were also collected at baseline (during pregnancy). Since maternal mood during the perinatal period may have a lasting influence on early caregiving and development, consideration of baseline depression as a predictor or covariate could provide important complementary insights. If baseline depression was excluded due to low prevalence or measurement timing, please state this explicitly and discuss the implications.<br>4. The Methods mention testing for interaction effects of HIV status and trial arm, but results are only briefly briefly presented ("There was no interaction by trial arm or HIV status", page 20, line 27). I suggest that the authors include both how interaction test was performed and the proper results for each test, thereby improving transparency.<br>5. Table 3 indicates that the association between maternal depressive symptoms and the MDAT fine motor score was marginally significant (p=0.065). This result deserves a brief comment in the Discussion, as it may suggest a subtle but potentially meaningful link between maternal mood and specific developmental domains. The authors might discuss whether this finding is consistent with prior literature suggesting that fine motor is particularly sensitive to variations in early maternal engagement.<br>6. The introduction could more explicitly articulate why maternal depressive symptoms might affect mother-child interaction in this specific context (mechanisms, previous mixed evidence in LMICs). This would strengthen the rationale for including depression as a key exposure.<br>7. The EPDS was administered at 24 months postpartum, long after the perinatal period for which the tool was designed. Please discuss the implications of this timing, e.g. does it capture chronic or current depressive symptoms? The low prevalence (≈5%) may partly reflect this issue. Additionally, given the low number of mothers meeting the EPDS cut-off, it is important to acknowledge potential under-powering to detect associations with OMCI or ECD outcomes.<br>8. It is unclear whether assessors of OMCI and MDAT/CDI were blinded to maternal EPDS scores or trial arm allocation. Please

	specify, as lack of blinding may bias observed associations.<br>Minor comments:<br>1. Use a single expression for the instrument's name throughout the text (e.g. Observation of Mother–Child Interaction (OMCI))<br>2. Depression prevalence is listed as 4.8% in the Abstract but 1.3% in Table 1. Please reconcile these numbers.

VERSION 1 – AUTHOR RESPONSE

**Comment**	**Reply**	**Page**
**Major comments**		
The Methods section mentions that a conceptual framework guided the identification of potential confounders (Supplementary Figure 1) and that variables were tested using bivariate models before inclusion in multivariable analyses. However, it remains unclear which covariates were actually retained in the final adjusted models shown in Tables 2 and 3. Although these variables are listed in the table footnotes, it would be preferable to describe them explicitly in the Methods section, along with a brief rationale for their inclusion. In addition, while the framework presented is conceptually sound, it appears to represent a conceptual rather than a formal causal model. The authors might consider discussing how a Directed Acyclic Graph (DAG) could be used to clarify hypothesised causal relationships between maternal depression, mother–child interaction, and ECD outcomes. This would help to distinguish true confounders from mediators or colliders and enhance the causal interpretability of the adjusted analyses.	We have added a description of the covariates in the Methods section alongside explanation of why they were included (through understanding of the factors that may be associated with the outcomes by drawing a framework).<br><br>Thank you - we have replaced our previous conceptual framework with a Directed Acyclic Graph to clarify the hypothesised causal relationships, of which there are three (maternal-child interaction -> ECD outcomes, maternal depressive symptoms -> ECD, maternal depressive symptoms -> maternal child-interaction). The DAGs are included in the Appendix.	13<br><br>32-34
The Coping Strategies Index is presented in Table 1 but not discussed elsewhere in the text. It would be useful to provide a brief description of how this index was constructed and scored, including the range of possible values, what higher scores indicate, and whether it reflects short-term or chronic food insecurity. Additionally, please explain whether this variable was considered as a potential confounder (e.g., proxy for household stress or food insecurity) in the adjusted analyses. Clarifying its role would improve readers' understanding of the socioeconomic context of the sample.	We have added a description of the Coping Strategies Index to the Methods with brief description. A list of potential confounders which includes the CSI has been added to the Methods, explaining that this was not included in the final adjusted analysis.	11
The paper focuses on EPDS measured at 24 months, yet depressive symptoms were also collected at baseline (during pregnancy). Since maternal mood during the perinatal period may have a lasting influence on early caregiving and development, consideration of baseline	We apologise for the lack of clarity regarding this. We have considered maternal depressive symptom exposure in our study as *EPDS >=12 at baseline OR*	9-10

depression as a predictor or covariate could provide important complementary insights. If baseline depression was excluded due to low prevalence or measurement timing, please state this explicitly and discuss the implications.	*24 months* and we have added clarification of this to the paper in the Methods.<br><br>We have also added this as a variable in the Baseline Characteristic Table to add further clarity.	 <br><br>18
The Methods mention testing for interaction effects of HIV status and trial arm, but results are only briefly briefly presented ("There was no interaction by trial arm or HIV status", page 20, line 27). I suggest that the authors include both how interaction test was performed and the proper results for each test, thereby improving transparency.	We have added explanation of how the interaction test was performed in the Methods section. The p- value from results of this analysis have been included in the Results section for the reader as suggested without including too much information in the main bulk of the paper.	14, 21
Table 3 indicates that the association between maternal depressive symptoms and the MDAT fine motor score was marginally significant (p=0.065). This result deserves a brief comment in the Discussion, as it may suggest a subtle but potentially meaningful link between maternal mood and specific developmental domains. The authors might discuss whether this finding is consistent with prior literature suggesting that fine motor is particularly sensitive to variations in early maternal engagement.	Thank you for the comment. The point estimate - 0.527 (-1.087 – 0.033) p-value 0.065 is not significant at the p<0.05 level and would represent a very small effect as Fine Motor domain is out of 36. We feel there is a high risk of type 1 error from multiple comparisons and we are particularly conscious that we do not want to suggest a finding which may be false positive.	
The introduction could more explicitly articulate why maternal depressive symptoms might affect mother-child interaction in this specific context (mechanisms, previous mixed evidence in LMICs). This would strengthen the rationale for including depression as a key exposure.	We have added more explanation of the existing evidence for this relationship and some of the suggested mechanisms, most importantly through the quality of responsive parenting.	6-7
The EPDS was administered at 24 months postpartum, long after the perinatal period for which the tool was designed. Please discuss the implications of this timing, e.g. does it capture chronic or current depressive symptoms? The low prevalence (≈5%) may partly reflect this issue. Additionally, given the low number of mothers meeting the EPDS cut-off, it is important to acknowledge potential under-powering to detect associations with OMCI or ECD outcomes.	The questions in the EPDS are screening questions for depressive symptoms and, despite the name of the tool, they are not specific to the postnatal period. We have referenced literature demonstrating that the EPDS can be used to assess current depressive symptoms in women with older children and also in general adults. We have also	9, 10, 25

	added the important potential for under-powering to our limitation section. Finally, we have clarified that the EPDS was administered at two time points: during pregnancy, and at 24 months postpartum.	
It is unclear whether assessors of OMCI and MDAT/CDI were blinded to maternal EPDS scores or trial arm allocation. Please specify, as lack of blinding may bias observed associations.	We have added clarification to the paper that assessors of OMCI and MDAT/CDI were blinded to trial arm allocation but not to maternal EPDS score, as these were collected by the research nurse at the same study visit. We do not feel this has introduced much bias to results as we had 87% (OMCI) and 94% (MDAT) percentage agreement in a proportion of assessments that were video recorded and double- marked by experts in the tools. The assessors were not aware of plans to explore associations between maternal depression and maternal-child interaction.	25
**Minor comments**		
Use a single expression for the instrument's name throughout the text (e.g. Observation of Mother–Child Interaction (OMCI))	We have checked carefully and believe we have now consistently used the term OMCI throughout the manuscript	21, 23
Depression prevalence is listed as 4.8% in the Abstract but 1.3% in Table 1. Please reconcile these numbers.	We apologise for the lack of clarity regarding this. The maternal depressive symptom prevalence refers to *EPDS>=12 at baseline OR 24 months*. In the baseline characteristic table we initially included this as separate variables but to provide clarity we have now added the bespoke variable in the Baseline characteristic table, explained the variable clearly in the Methods and rectified the values so they are consistent throughout the paper.	3, 9, 10, 18

VERSION 2 – REVIEW

**REVIEWER NAME**	Maruyama, Jessica Mayumi
**REVIEWER AFFILIATION**	Mackenzie Presbyterian University
**REVIEWER CONFLICT OF INTEREST**	No Conflict of Interest
**AI DISCLOSURE**	ChatGPT was used exclusively for English language revision and to enhance the clarity of the review text. All evaluative content, comments, and conclusions are the sole responsibility of the reviewer.
**DATE REVIEW RETURNED**	30-Mar-2026

**GENERAL COMMENTS**	Thank you for the careful revision and for the detailed responses to the reviewers' comments. The manuscript has improved substantially, and the authors have made a strong effort to address the main concerns raised in the first round. Overall, I believe this is a

	valuable study with clear scientific merit and important social relevance, and I support its acceptance.<br><br>For the sake of transparency in the open review record, I would like to briefly clarify two of my earlier comments, which I felt were only partially addressed, although not in a way that would justify a further round of revision. First, regarding the marginal association between maternal depressive symptoms and the MDAT fine motor score, my original suggestion was not intended to imply that this finding should be interpreted as statistically significant or overstated. Rather, I thought it could merit a brief and cautious mention in the Discussion as a potentially informative trend, especially if considered in light of previous literature on the sensitivity of specific developmental domains to maternal mood and engagement. I understand and respect the authors' conservative position, especially in light of the possibility of type I error due to multiple comparisons; however, my original point was that a careful mention of the result would not necessarily amount to overinterpretation.<br><br>Second, regarding the use of the EPDS at 24 months postpartum, my comment was not meant to question whether the instrument could be used beyond the immediate postnatal period, as there is indeed literature supporting its broader use as a screening tool for current depressive symptoms. Rather, I wished to highlight the importance of interpretive precision. At that time point, the EPDS is more likely to capture current or persistent depressive symptoms in mothers of older children than symptoms specific to the postpartum period itself, and I felt that making this distinction explicit would strengthen the conceptual interpretation of the findings. I also appreciate that the authors acknowledged the possibility of limited statistical power given the low prevalence of mothers above the EPDS cutoff.<br><br>These points, however, do not alter my overall positive evaluation of the manuscript. The revised version is clearly stronger, and I believe the study makes a meaningful contribution to the field.
